# Understanding “Childhood Poly-Victimization” to help uncover abuse during child investigative interviewing: a systematic review

**DOI:** 10.3389/fpsyg.2024.1395940

**Published:** 2024-09-16

**Authors:** Pantxika Victoire Morlat, Laurence Alison

**Affiliations:** Department of Psychology, Institute of Population Health, University of Liverpool, Liverpool, United Kingdom

**Keywords:** poly-victimization, child victim, child abuse, victimization, investigative interview, forensic interview, rapport, polyvictimization

## Abstract

**Introduction:**

It is well established that child victims are some of the most challenging populations to interview. Indeed, children tend to feel ashamed, scared or in denial, leading to difficulties for law enforcement when gathering information, and subsequently with prosecuting offenders. Moreover, with crimes against children increasing, it is common for interviewed victims to have experienced several abuses (poly-victimization). Thus, the main goals of this study are to (1) identify the facets that are included in poly-victimization, in order to (2) provide a clear definition that can be used by law enforcement during child interviews, which could lead to (3) a better identification of cases of abuse to maximize safeguarding and protect children.

**Materials and methods:**

A systematic review was used to establish the differences in conceptualizations of poly-victimization as well as the measurements chosen by research to measure this concept. The current systematic review included research articles on childhood poly-victimization using a quantitative measuring instrument published as of 2007, that focused on populations under 18 years of age.

**Results:**

The findings were divided into (1) conceptual dimensions, (2) characteristics of studies’ populations and (3) measures and psychometrics properties. It was found that research used various terms for poly-victimization, yet with numerous definitions implying differences in number of abuses, time frame, and mental health aspects to be considered in the identification of poly-victimization. The included papers (*n* = 6) were from Europe, Asia, and the United States. Over half of the studies used the Juvenile Victimization Questionnaire (JVQ) but adapted either (i) to the study’s population, (ii) possible answers, (iii) wording of questions, or (iv) by the removal of questions.

**Conclusion:**

Our findings highlight the need for a more theoretically coherent definition of childhood poly-victimization. Questions regarding number of abuses, time consideration (past year vs. lifetime), mental health and severity of abuse should be addressed to develop a unified definition of poly-victimization. Rapport-based interviews should be the focus to uncover the truth and to avoid a secondary trauma in the crisis of a child’s disclosure of abuse. A new definition of “Childhood Poly-Victimization” is proposed by the authors as well as a “Decision Tree for Identifying Childhood Poly-Victimization” designed to be used by law enforcement during child investigative interviews.

## 1 Introduction

During their lifetime, children and adolescents generally experience victimization in four or more environments (e.g., family, school; Pereda et al., 2014; Morlat et al., 2022). In a sense, it can be assumed that one victimization generally leads to another (Pereda et al., 2014; Hébert et al., 2018). This accumulation of victimization had led to the development of a concept called *poly-victimization*, which refers to the exposure to different kinds of victimization in different settings (Finkelhor et al., 2007). In a study by Pinto-Cortez et al. (2018), 89.1% of adolescent respondents reported being poly-victims. Furthermore, according to The National Survey of Children’s Exposure to Violence used by Finkelhor et al. (2011), 38.7% of children reported experiencing at least one incident of victimization (either direct or indirect). Of those children, 10.9% reported five or more direct exposures to different types of violence and 1.4% reported 10 or more direct victimizations. Notably, poly-victims are two to seven times more likely to be victimized in the future and are four to six times more likely to experience another victimization in the next year than non-poly-victims (Finkelhor et al., 2009). Due to the seriousness of what poly-victimization entails, recent focus on this concept and what it encompasses has hatched from various fields, including Psychology. As the number of victims has increased over the past years [partly due to the pandemic and the recognition of new cyber crimes; Office for National Statistics (ONS), 2020; The National Society for the Prevention of Cruelty to Children (NSPCC), 2022; Home Office, 2023], the demand for investigations to aid victims of abuse has increased simultaneously.

Since children are defined as “vulnerable” due to their ages, they require adapted measures that are effective for this group (Ministry of Justice, 2022). Finding “effective” measures involves adopting techniques that are successful when deployed in the field for a particular purpose and with a specific population (Wilson and Lipsey, 2001). Prior research in Psychology have assisted in the development of instruments for law enforcement practice in the context of child protection (see Long et al., 2016) and investigative interviews (see Alison et al., 2013). Moreover, multiple guidelines such as the Achieving Best Evidence (ABE) guidelines, Special Measures guidelines (Ministry of Justice, 2022), the National Institute of Child Health and Human Development protocol (NICHD; developed by Lamb et al., 1998), then revised NICHD protocol (RP, as advised by Hershkowitz et al., 2014), were created to improve children’s ability to talk (Ministry of Justice, 2022). However, some of these guidelines have shown to be inconsistent with new research in the field of communication Psychology, developmental Psychology and forensic Psychology which are essential in the improvement of the effectiveness of investigative interviews with children (e.g., divided attention decreases false memory in children; Otgaar et al., 2012). Children tend to be both victims and witnesses (Johansson et al., 2018), which makes the focus on improving existent guidelines, as well as creating new guidelines specific to children, a core focus for efficient practice. Indeed, the information gained during child interviews plays a central role in the investigation, and the progress of the police investigation generally depends upon (a) the child’s account of the incident and (b) the interviewer’s ability to obtain information from the child. To note, there was a 106% increase in child cruelty and neglect offenses in England over the past 5 years (The National Society for the Prevention of Cruelty to Children, 2023). Child victims are more vulnerable to suggestive interview strategies, confusion arising from complex questioning, and a lack of support during interviews (Goodman et al., 2014). Despite their age, children are likely to be aware of the judicial and intra-familial consequences of their allegations (Niehaus et al., 2017), which has to be overcome by the right amount of support provided by law enforcement forces. The College of Policing provide training in investigative interviews with children. However, the police still report difficulties in obtaining accurate accounts during child interviews (Cassidy et al., 2020), and in conducting efficient rapport-based interviews (Kim et al., 2020). In the context of investigative interviewing, Abbe and Brandon (2014) define rapport as positive interpersonal interactions that can enhance trust of the witness towards the interviewer and thus increase cooperation. Therefore, due to the increased number of child abuse cases, there is an increased need to study poly-victimization, and to include it in the current victim interview guidelines to make sure that the victims are well-identified (from victim to poly-victim). Thus, the principal aim of the study is to understand the differences in definitions of “poly-victimization” for a better identification of child poly-victims during police investigations. This paper will explore the concepts and measurements of child poly-victims through a systematic review.

It has been found that poly-victimization causes both short and long term morbidity and even mortality due to extreme violence in school and/or familial environment (e.g., sexual abuse), and even self-injured violence such as suicide (Finkelhor et al., 2007; Finkelhor et al., 2013). Even though being exposed to violence in a single context is traumatic, poly-victimization leads to severe impacts on mental health, in addition to emotional, developmental and behavioral problems (Cyr et al., 2014; du Plessis et al., 2015). This has practical implications in a forensic context, such as the behaviors of poly-victimized minors when questioned about abuse. According to Pereda and Gallardo-Pujol (2011), multiple mistreatments can lead to irreversible neurobiological changes, specifically to the hypothalamic–pituitary–adrenal neuroendocrine axis, part of the brain involved in stress feeling. Again, the latter is relevant to investigative interviews, interviewers should be aware of the cognitive, emotional and physiological consequences of multi-abuses during an interview (e.g., child shaking as a stress response when asked to recall events). Cognitive and emotional differences between children and adults can lead children to adopt “non-normal” victim behavior (Summit, 1983), this differs from entrenched beliefs about how victims should behave in the interview process, and can lead to children sometimes being stigmatized as liars or manipulators. Being aware of the reasons why a child may not react the expected way to trauma, or will not adopt a coping behavior typically held by adults, is essential to comprehend children during investigative interviewing. Overall, victimization leads to undeniable repercussions, but the identification of poly-victims may differ according to countries.

Differences in cultural norms as well as divergent legal justice systems shape the concept of poly-victimization between countries (Musicaro et al., 2019); hence influencing the methods and instruments used to measure childhood poly-victimization. Poly-victimization is defined in various ways, as The National Child Abuse and Neglect Technical Assistance and Strategic Dissemination Center (CANTASD) stated “There is no clear consensus around the definition of polyvictimization, the term describes the collective experiences of multiple types of violence, usually in multiple settings, and often at the hands of multiple perpetrators” (The National Child Abuse and Neglect Technical Assistance and Strategic Dissemination Center, 2018, p. 1). Some identified child poly-victims by the number of different types of victimization they experienced (Finkelhor et al., 2009), others have considered child victims experiencing poly-victimization if they reported four or more unique types of victimization in the past (Pereda et al., 2014), or sometimes in the past year (Emery et al., 2022). Another method is to assume that poly-victimization increases with age, and therefore group cohorts into chronological age assuming that older children experience more victimization (Dierkhising et al., 2019). Those are crucial aspects to recognize when considering that definitions shape the criteria to define mental disorders. For instance, some researchers have included witnessing “trauma” as part of the measure of poly-victimization in order to meet the *Diagnostic and Statistical Manual of Mental Disorders* (*DSM-5*) criteria for post-traumatic stress disorder (PTSD; Ford et al., 2013; Grasso et al., 2015). Moreover, according to The National Child Abuse and Neglect Technical Assistance and Strategic Dissemination Center (2018), researchers differentiate *multiple victimization* and *poly-victimization*, arguing that poly-victimization is a form of multiple victimization. Arguments are based on the belief that individuals who experience various types of victimization have higher levels of trauma symptoms than those who experience one same type of victimization (Turner et al., 2010; Hamby et al., 2014). According to this theory, it could be suggested that a 12-year-old who has been experiencing physical abuse and bullying by her peers as well as witnessing violence at home may feel more traumatized than a 12-year-old who has been sexually abused for 6 years by a family friend.

Despite grounded research on the topic, concepts remain questionable in their validities and more research is needed to develop and validate unified definitions and assessments of “childhood poly-victimization” and the cumulation of traumatic exposure. This would not only be beneficial to understand what poly-victimization means and encompasses, but also to identify the most effective ways to measure and subsequently prevent poly-victimization and its impacts. In sum, despite the focus of scientific research on the concept of poly-victimization for the past 15 years, the definitions and theoretical framework still lack clarity and consistency, impacting on the effectiveness of protecting children.

The purpose of the study is to determine a more theoretically coherent concept of defining childhood poly-victimization with the aim of developing a better poly-victimization conceptual model to be used effectively in research, response to, and prevention of this phenomenon. Overall, the main goals of this study are to (1) identify the facets that are included in poly-victimization, in order to (2) provide a clear definition that can be used by law enforcement during child interviews, which could lead to (3) a better identification of cases of abuse to maximize safeguarding and protect children.

## 2 Materials and methods

### 2.1 Search strategy

The research on poly-victimization should consider a child-centered approach. This means, when studying and measuring victimization, researchers should consider child populations in order to (i) test the capacity of minors to understand the concept of being poly-victims, and (ii) in case of disclosure, provide the adaptive help to prevent the abuse from continuing. Hence, the current systematic review identified scientific papers that focused on populations under 18 years of age on both (1) the chosen instrument and (2) the conceptual background/definitions of poly-victimization. Several databases were reviewed, using key terms “childhood poly-victimization/polyvictimization*, *child victimization*, *children victimization*, *children poly-victimization*, *adolescent polyvictims*, *children polyvictims*, *children victim of abuse*, *adolescents victimization*, *minors polyvictims*.” Three main databases used to carry out the research were: ScienceDirect, PubMed, and Web of Science as those were the favored databases for the field of Psychology, and other databases (e.g., Scopus) included the same articles as the chosen three. Combinations of these conditions were carried out and filtered by Title, Abstract, and Keywords. After reviewing the results of the above keywords, the authors chose *childhood poly-victimization* [and *childhood polyvictimization* to avoid the exclusion of papers using the American spelling] as the favored keyword. The choice of solely assessing articles with the use of “poly-victimization” or “polyvictimization” was based on the aim of evaluating what authors interpret as poly-victimization and whether a consensus on its definition has been established.

### 2.2 Inclusion and exclusion criteria

The systematic search was restricted to research articles published from 2007, as the first conceptualization of “polyvictimization” by Finkelhor et al. (2007) was developed that year, to October 2023 (month of the data collection). Thus, the inclusion criteria referred to (1) studies published as of 2007; (2) research articles; (3) studies on childhood poly-victimization using a quantitative measuring instrument; (4) studies on minor populations (below the age of 18); and (5) studies published in English or French (the lead author being bilingual in both languages). The exclusion criteria referred to (1) studies published before 2007; (2) documents other than research articles; (3) studies not including poly-victimization/multiple victimization measures; (4) studies on adult populations; and (5) studies written in languages other than English or French.

### 2.3 Data coding

**Figure 1:**
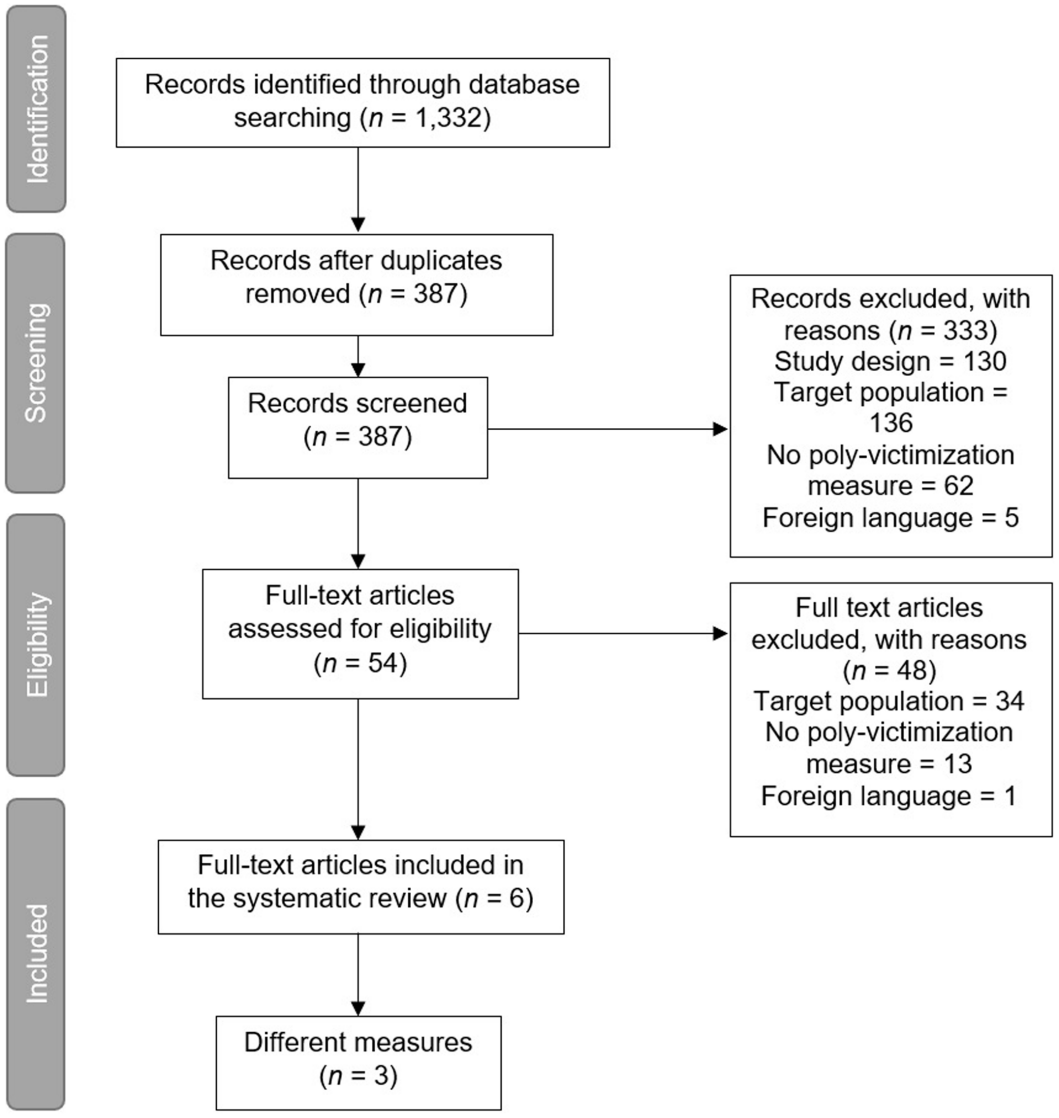
Documents selection diagram.

Firstly, an analysis was performed on the theoretical dimensions, their definitions, and items’ content. Secondly, the authors coded the countries where the measures were administered, and the characteristics of the sample in terms of age. Thirdly, an analysis of the instruments’ properties was run, the names of the new instruments were reported, and in case of adaptation, the references of the original ones were provided. See Figure 1 for Preferred Reporting Items for Systematic Reviews and Meta-Analyses (PRISMA; Moher et al., 2009) diagram.

### 2.4 Quality assessment

The Standard Quality Assessment Criteria for Evaluating Primary Research Papers from a Variety of Fields (SQAC; Kmet et al., 2004) was used for quality assessment. The SQAC contains separate point-based checklists for quantitative methodologies. The SQAC checklist was used to assess the quality of quantitative studies. Any discrepancies between the study’s authors were discussed, and quality assessment proposed suggestions for future research.

## 3 Results

### 3.1 Theoretical dimensions

All selected studies interpreted poly-victimization as an accumulation of victimization, however, conceptual definitions differ between research and inclusion/exclusion of some aspects vary. Firstly, the notion of mental health was included by some researchers, suggesting that poly-victimization may *lead* to mental health issues, or may be *felt* as victimizations. For instance, Pereda et al. (2014, p. 640, 647) defines the concept of poly-victimization as multiple victimization experiences in different episodes—this is unclear whether by “different episodes,” the authors mean at different times, or episodes of one’s life (could be 3, 6 months, or a year)—with an “effect on a *child’s wellbeing*”. This is interesting considering that poly-victimization has been found to deteriorate mental health, therefore, it is wise to wonder if Pereda et al. (2014) suggests that mental health is a core component of poly-victimization or that mental-health and poly-victimization are associated concepts. Other studies have included the notion of “trauma” as part of their definitions of poly-victimization. Indeed, Cuevas et al. (2009, p. 639) adds “traumatic” victimization in their conceptual interpretation while Connell et al. (2018, p. 525) alters between poly-victimization and multiple potentially *traumatic* event exposures suggesting that those are interchangeable concepts. Similarly, Emery et al. (2022, p. 24) includes depression in the explanation of poly-victimization by adding that “depression may be more profound for those adolescents who have suffered from multiple forms of maltreatment (also known as polyvictimization).” Not only do Cuevas et al. (2009) and Emery et al. (2022) share similar thoughts on the link between mental health and poly-victimization, but both papers also suggest that the concept of *multiple forms of maltreatment* shares the same approach as poly-victimization. Both argue that poly-victimization and multitype maltreatment encompass the same notion of “multiple different forms of victimization and/or abuse experiences” (Cuevas et al., 2009, p. 640) or “multiple forms of maltreatment” (Emery et al., 2022, p. 22428). By emphasizing on “multiple,” the authors suggest that poly-victimization includes different forms of abuse. However, it is unclear what it meant by “forms of maltreatment” and whether this has to occur more than once for each “form” to be classified as poly-victimization, or one form of maltreatment has to occur but if this happened multiple times then it could also be defined as poly-victimization. Here, this aspect on *time* is suggested but not clearly defined, whereas other authors have taken *time* as a core dimension of poly-victimization. Indeed, Jackson-Hollis et al. (2017) defines poly-victimization as a “multitude of different types of victimization on *many different occasions*” (p. 350), which according to this statement, implies that a victim who experienced multiple forms of victimization on a single-occasion may not be defined as poly-victim. The notion of time is also suggested by Pereda et al. (2014) whose interpretation of poly-victimization includes “different episodes.” Accordingly, Connell et al. (2018, p. 519) asserts that poly-victimization occurs so frequently that “it is the norm for victimized children”. This latter evokes the sense that poly-victims experience victimization very regularly, however, it is unclear if (a) the same type of victimization must be experienced regularly, or (b) different types of victimization must be experienced regularly, or (c) if the same type of victimization/different forms of victimization experienced not so regularly could be still considered poly-victimization. Zhang et al. (2021) further develops this idea, by emphasizing on the aspect that one particular form of adversity, even when serious and repeated, may have less harmful impacts than multiple forms of adversities (defined as poly-victimization by Zhang et al., 2021). In sum, this could be interpreted that different forms of adversities when experienced once would have higher negative impacts than one form of victimization experienced 20 times. On another note, Jackson-Hollis et al. (2017) specified that poly-victimization could be a consequence of the same or different perpetrator.

### 3.2 Characteristics of studies’ populations

#### 3.2.1 Country

The instruments used to measure poly-victimization were administered in Europe, the United States, and Asia. Specifically, in European countries such as Spain (*n* = 1) and the United Kingdom (*n* = 1). Few of the studies focused on North American population (United States, *n* = 2). Asia was represented by two papers, from China (*n* = 1) and Nepal (*n* = 1).

#### 3.2.2 Population type (age)

One of the studies in the United States had the largest range of age as the participants were between 2 and 17 years old at the time of the research (Cuevas et al., 2009). The other research in the United States included participants between 7 and 17 years of age (Connell et al., 2018). In European studies, the selected study in Spain had a range of 12-17 year old participants (Pereda et al., 2014), while in the United Kingdom, the range was smaller (between 13 and 16 years old; Jackson-Hollis et al., 2017). Studies in Asia had similar range of ages as the North American and European studies as the participants were between 8 to 14 (Zhang et al., 2021) and 11 to 16 years old (Emery et al., 2022).

### 3.3 Measures and psychometrics properties

Despite research evaluating/measuring the prevalence of childhood poly-victimization, different measures were adopted to evaluate the same concept, even in research conducted in the same country. Firstly, it is important to note that out of the six selected studies, four of them used the Juvenile Victimization Questionnaire (JVQ) developed by Finkelhor et al. (2005); however, each of the studies adapted the JVQ to match their conceptualizations of poly-victimization and their opinions of the adaptive way of measuring it. For instance, Jackson-Hollis et al. (2017) chose the JVQ to measure poly-victimization but used an adapted JVQ version for a United Kingdom sample (Radford et al., 2013) which excluded questions regarding intrafamilial victimization. A total of 24 screener questions were asked from the adapted JVQ, plus two additional questions on internet and mobile phone victimization from the National Survey of Children’s Exposure to Violence (NatSCEV; Finkelhor et al., 2009). Accordingly, Pereda et al. (2014) adopted a Spanish version of the JVQ, this included the translation of the questionnaire in Spanish and Catalan, but also the removal of an item to fit with Spanish law. Indeed, the item “statutory rape” was removed because the authors estimated that it was not relevant to the values and social standards in consensual sexual relationships in Spain (in 2022, Spanish law on sexual abuse defined rape as “sex without consent”; Burgen, 2022; Gregory, 2022). Also, similarly to Jackson-Hollis et al. (2017), Pereda et al. (2014), included two new items related to electronic victimizations to evaluate virtual harassment of minors. In total, 36 forms of victimization classified into six domains were asked to minors: (1) conventional crime, (2) caregiver victimization, (3) victimization by peers and siblings, (4) sexual victimization, (5) witnessing and indirect victimization, and (6) electronic victimization. Similarly, Cuevas et al. (2009) excluded the item on “statutory victimization” for children under 12 years old (participants age range between 2 and 17 years old). Furthermore, they excluded the item “dating violence” for minors under 12. Out of the six articles, Emery et al. (2022) also chose the JCQ to measure poly-victimization. However, as Cuevas et al. (2009), Pereda et al. (2014), and Jackson-Hollis et al. (2017) adapted the JCQ, Emery et al. (2022) modified the JCQ so that the possible responses were (1) happened in the past year, (2) happened but not in the past year, and (3) never happened. The JCQ item on physical abuse by parents/caregivers (“not including spanking on the bottom…did a grown-up in your life hit, beat, kick, or physically hurt you in any way”) was used to create a “Ever physically abused” dummy variable indicator, moreover, the four JCQ items on child sexual abuse (“there was sexual contact with an adult you know,” “sexual contact with an adult you do not know,” “a child your age made you do sexual things,” and “anyone tried to force you to have sexual intercourse”) were combined into a “Ever sexually abused” dummy variable. And the item “were you neglected” was changed into a “Ever neglected” dummy variable. Nevertheless, despite using the same instrument as the basis on their analyses, Emery et al. (2022) analyzed poly-victimization as two or more victimizations, Jackson-Hollis et al. (2017) stated that poly-victims were more likely to experience three to four victimizations, while Pereda et al. (2014) defines participants who experienced 1–3 forms of victimizations as the “victimization group,” 4–6 as the “low poly-victimization group” and seven to more as the “high poly-victimization group.” Interestingly, alike Cuevas et al. (2009), the study by Connell et al. (2018) was in the United States, however the instrument used to measure poly-victimization was different. Connell et al. (2018) used the Trauma Exposure Screen (THS; Lang and Franks, 2007) which measures the lifetime frequency of exposure to 19 different Potentially Traumatic Events (PTEs). An aspect of this tool is the subjective level of distress related to each. The THS is based on five-point Likert scale from zero (never) to four (10 or more times). The higher the rates of exposure to PTEs, the higher the poly-victimization was rated (high, moderate, low). However, the fact that poly-victimization can be classified based on the victimization might be difficult, and may not reveal reality (children may not have reported more “victimizations”, leading them to be identified as low poly-victims). Similarly, Zhang et al. (2021) chose a different way to measure poly-victimization. Indeed, childhood maltreatment was rated either by “0” not present, “1” occasionally present/took place on one occasion, and “2” frequently present/took place on multiple occasions. Poly-victimization was defined as experiencing two or more types of victimization. Zhang et al. (2021) believed that this cut-off of “2” captured true incidences of adversity that were most likely to have impacted mental health, thus, more likely to be recalled accurately. Experiencing one type of victimization that was coded as “2” was used to categorize as “re-victimization,” those who suffered more than one episode of one type. Therefore, “re-victimization” and poly-victimization were conceptualized as two different concepts. The efficacy of this hyper-specific approach during the investigative interview process could be questioned, as obtaining this detail may do little to improve efficiency of the interview process.

## 4 Discussion

### 4.1 Main findings

Research on poly-victimization differ in their conceptualizations, definitions, and measurements. Theoretical dimensions changed depending on the measures taken by the authors, illustrating a non-consensual definition of poly-victimization. Rightly so, it could be assumed that countries share different values, but it was even found that research in the same country (United States) differ in both their definitions and measures of poly-victimization (Cuevas et al., 2009; Connell et al., 2018). This undeniably has an effect on the comprehension of the issue that children experience, which subsequently leads to misconceptions or alternative interpretations of what makes a child a victim of poly-victimization. For instance, some authors include trauma in their conceptualizations of poly-victimization (Ford et al., 2013; Grasso et al., 2015), however, if a child is not defined as a poly-victim, their feelings of trauma may not be considered as such which could lead to a non-diagnosed PTSD. The hyper-specialization in the field of Medicine, Sociology, Psychology and public health limits the conceptual understanding of *victimization*, which impacts the effort to fight societal issues (Hamby et al., 2014).

### 4.2 Interpretation of findings

Before discussing on the divergent definitions of poly-victimization, it is important to note that some authors study poly-victimization and “multiple traumatic events” as interchangeable concepts. For instance, Connell et al. (2018) alter between multiple PTEs and poly-victimization, which implies that each victimization can potentially be traumatic for children. By looking at the measure that Connell et al. (2018) chose, it is clear that the authors interpret poly-victimization as a form of trauma since the THS (which measures the lifetime frequency of exposure to PTEs) was used to measure poly-victimization. Similarly, Emery et al. (2022) suggest that “multiple forms of maltreatment” (p. 22429) and poly-victimization are interchangeable concepts arguing that both refer to the multiple forms of abuse experiences. Notably, it is important to note that according to the Cambridge Dictionary, “poly” and “multi” do not refer to the exact same concept. Indeed, “poly” from the Greek *polys* meaning “much” refers to an abundance of something; while “multi” from the Latin *multus* meaning “many” refers to “strong, great.” In a sense, “poly” refers to the accumulation of something while “multi” defines more the adversity of something. The authors argue that “poly-victimization” and “multiple victimizations” may differ in their concepts, while poly-victimization suggests an accumulation of victimization (the addition of different types of victimizations?), “multiple victimizations” emphasizes on the severity of the victimizations (the number of times the victimizations occurred?). The measure chosen by Emery et al. (2022) supports these differences in meanings, indeed, they adapted the JCQ so that possible responses were divided into (1) happened in the past year, (2) happened but not in the past year, and (3) never happened. This maintains the concept that *multi* refers more to the adversity/number of times the abuse occurred, in the reasoning of Emery et al. (2022) in their interchangeable notion of “multiple forms of maltreatment” and poly-victimization. However, because of the complexity to identify what makes a child poly-victim (Emery et al., 2022) and how many PTEs should occur to be considered a form of poly-victimization (Connell et al., 2018), the measure chosen remains unclear in some aspects, questioning the inclusion of time in the definition of poly-victimization.

It is important to note that the two studies discussed above were from two different countries (Nepal and United States) despite their similar interpretations of poly-victimization (Connell et al., 2018; Emery et al., 2022). Studies from the same country (United States) have similarly interpreted poly-victimization by including a sense of trauma to it (Cuevas et al., 2009; Connell et al., 2018). However, despite similar conceptualizations and potentially shared cultural values, the chosen instruments were different. As mentioned, Connell et al. (2018) used the THS while Cuevas et al. (2009) used an adapted version of the JCQ excluding items related to dating violence and statutory victimization for children under 12 (age range for this study being 2–17 years old). Both papers supported the inclusion of trauma in their definitions of poly-victimization but only one included it in their measures (THS; Connell et al., 2018). Despite evident impacts on mental health, this questions the validity of including trauma in the definition of poly-victimization. Indeed, it is possible that some children may not realize that they are “traumatized” either because they (i) do not recognize the symptoms of trauma, (ii) have normalized the abuse, (iii) are in denial of the abuse, (iv) do not want to be diagnosed as having mental health issue, or (v) the presence of caring adults mitigates the sense of trauma. Arguing against the symptoms of PTSD may be a form of fight against the occurrence of the non-consented abuse, which leads to question the inclusion of trauma (and other psychological impact) in the definition and measure of poly-victimization.

Notably, Cuevas et al. (2009) used the JCQ for the measure of poly-victimization with no additional questions, whereas other authors using the JCQ added questions about new forms of victimization that the JCQ does not include since its appearance in 2005. Despite adaptations of the JCQ to different countries (e.g., Spain and United Kingdom), both Pereda et al. (2014) and Jackson-Hollis et al. (2017) included questions related to electronic victimizations (e.g., mobile and internet victimizations). This addition seems sensible since there has been an increase of crimes on the Internet—for instance, online grooming crimes have risen by more than 80% in 4 years (The National Society for the Prevention of Cruelty to Children, 2022). However, it is important to mention that all studies may not modify the JCQ by changing/adding variables, especially since this would require additional statistical tests to measure its reliability. Therefore, a revised version of the JCQ may be needed for the future, in accordance to changes in psychological, forensic, and public policy domains.

Finally, both Pereda et al. (2014) and Jackson-Hollis et al. (2017) define poly-victimization as multiple victimization on different occasions, Jackson-Hollis et al. (2017, p. 350) specified “different types of victimization on many different occasions,” which implies that a child who experiences a single incident of different victimizations may not be considered a poly-victim. In Pereda et al.’s (2014) study, children who had experienced one to three victimizations during the past year were not considered “poly-victims” therefore excluding victimizations that occurred during their lifetimes. Zhang et al. (2021) develop this idea in distinguishing “re-victimization” and poly-victimization; indeed, they believe that one victimization which is severe and repeated has less impacts than multiple forms of abuse, defined as poly-victimization by Zhang et al. (2021). This was illustrated by the measure chosen by Zhang et al. (2021), in which poly-victimization was rated as different victimizations that were frequently present/took place on multiple occasions while re-victimization was rated as one form of victimization that was frequently present/took place. Notably, as pointed by Hamby et al. (2014), poly-victimization and “poly-perpetration” have been studied in isolation from re-victimization. These differences in measure and definition may result in blurry definitions that slows down scientific progress by not adopting an internally consistent terminology. Therefore, the authors propose a definition in regards to the findings of the current study.

Childhood poly-victimization: when an individual under the legal age of adulthood has been victimized more than once in their lifetime by the same or a different perpetrator, regardless of whether the type of abuse differs from the first abuse, and of whether or not the environment matches that of other experienced abuse(s).

### 4.3 Strengths, limitations, and future directions

The quantity of included papers limited the ability to form firm conclusions. Especially, to meet study aims, one of the criteria required papers including a population of minors (under 18), which limited the evaluation of the conceptualizations and measurements of poly-victimization.

Despite the included studies being from different countries, awareness of differences in other cultures depending on their norms, values and justice system is essential. The current study’s findings may be biased by the authors’ western cultural background, which limits transferability and generalizability of findings.

Future studies should consider what instruments were used to measure multiple forms of maltreatment in research that did not use the term “poly-victimization.” Also, it would be interesting to measure the reliability of the JCQ for contemporary research. The need of adapting the JCQ may be the focus of future research.

### 4.4 Practical implications

**Figure 2:**
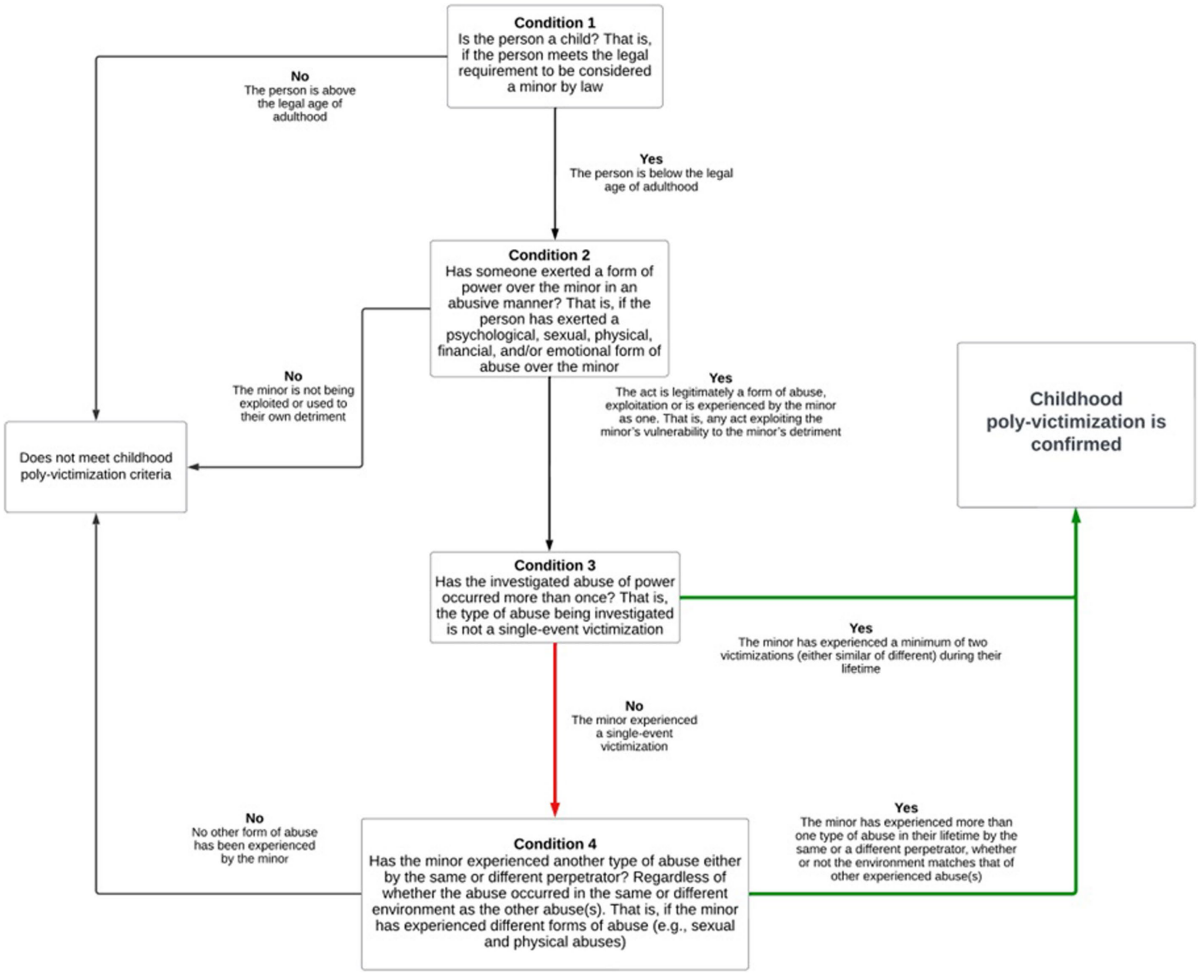
Decision tree for identifying childhood poly-victimization.

The World Health Organization (2021) has estimated that one in seven 10–19-year-old experience mental health disorders, accounting for 13% of the global burden of disease in this age group. Victimization of children likely contributes to these rates of mental health issues, emphasizing the importance of avoiding further victimization of child victims during the interview process by law enforcement, to prevent further compounding of trauma and mental health problems. Pointing out the differences of interpretations and measurements demonstrates inconsistencies in the conceptualizations of poly-victimization, and in what makes children poly-victims. This lack of clarity may lead to inconsistency in the actions aimed to help vulnerable children due to the absence of a stable framework. Developing consistent definitions of poly-victimization is not only profitable to research and knowledge, but also in prevention efforts, legal principles and framework, policy responses, and the formation of social norms. See Figure 2 for the “Decision Tree for Identifying Childhood Poly-Victimization” (lead author’s suggestion) aimed to be used by law enforcement during child interviews.

### 4.5 Conclusion

To conclude, this systematic review attempted to gather known information about the different concepts and instruments used to measure poly-victimization during childhood. Being aware of the facets of poly-victimization, and of how to identify a poly-victim is essential to comprehend and correctly identify child poly-victims during investigative interviewing. Indeed, including a clear definition of poly-victimization may produce more confidence in interviewers in identifying cases of abuse and thus increase effective practice. Furthermore, some children may be more reluctant to discuss certain type of abuse occurring at home (e.g., of sexual nature) but may feel less “ashamed” of discussing abuse at school (e.g., bullying). Therefore, knowing what poly-victimization means could open doors to developing rapport-building by improving the understanding that most victimized children are experiencing more than one abuse. Also, children may be interviewed for a specific suspicion of abuse, but by understanding the facets of poly-victimization, other forms of abuse could be detected by law enforcement. This can only be executed with efficient rapport interactions between the interviewers and interviewees, so that children feel comfortable enough to disclose abuse. The heterogenous definitions of poly-victimization, suggest that the concept of poly-victimization has not reached a consensus. It is wise to question whether “severity of the abuse” should be an aspect to include in the definition since such a classification of victimization seems complicated and ethically challenging. Additionally, “time consideration” in the definition of poly-victimization needs to be better defined as it is unclear whether one form of victimization that happens multiple times falls into the concept of poly-victimization. Furthermore, mental health outcomes may better fit into the concept of poly-victimization as its consequences rather than components of it. Lastly, clarity is needed regarding the definitions and measurements of the questions on re-victimization, poly-victimization, poly-perpetration and multiple victimization, and whether it is effective to identify them separately. Indeed, research assert that two forms of victimizations do not necessarily classify as poly-victimization (Pereda et al., 2014; Jackson-Hollis et al., 2017) while others maintain that it is sufficient (Emery et al., 2022). From the moment children have been victimized more than once, it could be assumed that they are poly-victims, not only to simplify analysis in research, but also to avoid minimizing the importance of each victimization. The refusal to change bad practice, coercive behaviors or pressure during investigative interviews involving children could leave them with few hopes of outside credibility and acceptance. In order to stop perpetuating the prejudicial myths behind child abuse, rapport-based interviews should be the focus to uncover the truth and to avoid a secondary trauma in the crisis of a child’s disclosure of abuse. The authors hope that this systematic review will help in the adoption of a unified definition of poly-victimization that can be used by law enforcement to identify cases of abuse during child interviewing (see Figure 2, lead author’s suggestion “Decision Tree for Identifying Childhood Poly-Victimization”).

## Data availability statement

The original contributions presented in the study are included in the article/supplementary material; further inquiries can be directed to the corresponding author.

## Author contributions

PVM: Conceptualization, Data curation, Formal analysis, Investigation, Methodology, Project administration, Resources, Software, Visualization, Writing – original draft, Writing – review & editing. LA: Investigation, Methodology, Project administration, Resources, Supervision, Validation, Writing – review & editing.
